# A Rare Case of Giant Cell Arteritis After the Administration of Checkpoint Inhibitor Therapy in a Metastatic Renal Cell Carcinoma Patient

**DOI:** 10.7759/cureus.50121

**Published:** 2023-12-07

**Authors:** Marjan Khan, Abdul Subhan Talpur, Chady Abboud Leon

**Affiliations:** 1 Internal Medicine, Marshfield Medical Center, Marshfield, USA; 2 Medicine, Liaquat University of Medical and Health Sciences, Jamshoro, PAK; 3 Hematology and Medical Oncology, Marshfield Clinic Health System, Marshfield, USA

**Keywords:** rheumatoid, polymyalgia rheumatica, mrcc, immune checkpoint inhibitors, giant cell arteritis, arthritis

## Abstract

We present a rare case of metastatic renal cell carcinoma in a patient who developed giant cell arteritis (GCA) after the administration of checkpoint inhibitor therapy with nivolumab and ipilimumab. The patient was initially treated with a combination of nivolumab and ipilimumab, showing a near-complete response with minimal side effects. However, after several cycles of checkpoint inhibitor therapy, the patient developed symptoms consistent with GCA, leading to a halt in the immunotherapy. This report highlights the complexity of managing the adverse effects of immunotherapeutic agents and the importance of a multidisciplinary approach in the management of complications such as GCA.

## Introduction

Renal cell carcinoma, a form of kidney cancer that arises from the proximal convoluted tubule lining which is responsible for transporting primary urine through the kidney’s small tubules, is prevalent in adults and accounts for around 90-95% of all cases of kidney cancer [1]. Metastatic renal cell carcinoma (mRCC) denotes the primary renal cell carcinoma’s diffusion from the kidney to other body organs [1]. mRCC exhibits varying characteristics and has an unsatisfactory five-year overall survival rate of 14% [2].

Immunotherapy medications known as checkpoint inhibitors have been developed to selectively activate T-cell regulatory pathways, thereby improving the immune response against cancer. As a result, these drugs have become a crucial component of medical interventions for various types of malignancies, such as bladder, colon, head and neck, Hodgkin lymphoma, liver, lung, renal, melanoma, mesothelioma, and stomach cancers [3]. Checkpoint inhibitors have demonstrated potential for specifically managing mRCC [3]. The available data underscores the assurance of utilizing checkpoint inhibitors as an innovative method of managing mRCC.

According to Ghatalia and colleagues, the use of checkpoint inhibitors in treating renal cell carcinoma has brought about a significant shift in conventional therapeutic approaches. Various inhibitors have demonstrated favorable outcomes and enhanced survival rates in clinical trials [4]. Tung et al. underscored the significant enhancement observed in clinical advantage and longevity when utilizing immune checkpoint inhibitors as the primary course of treatment for mRCC [5].

The utilization of immune checkpoint inhibitors in cancer therapies has substantially risen. Nevertheless, current studies have reported that it may produce unintended inflammatory consequences recognized as immune-related adverse events (irAEs), encompassing polymyalgia rheumatica with or without giant cell arteritis (GCA) [6,7].

Although the adverse effects of these therapies are being understood and treated more effectively, infrequent connections such as those between GCA and post-therapy underscore the significance of being more watchful. These occurrences emphasize that managing patients is about ensuring therapeutic effectiveness and weighing potential severe side effects. This account focuses on one specific episode to highlight the significance of a comprehensive and collaborative approach when addressing unforeseen difficulties in treating mRCC.

## Case presentation

A 74-year-old male presented with progressive weakness, fatigue, abdominal pain, weight loss, and dissatisfaction with previous work-ups at other healthcare centers. His past medical history included bilateral pulmonary emboli, for which he was on apixaban, coronary artery disease, for which a coronary artery bypass graft was done, hypertension, gastroesophageal reflux disease (GERD), and benign prostatic hyperplasia. He had new-onset hematuria, a 35-pound weight loss since May, low-grade fevers, sweats, right-sided abdominal pain shifting occasionally to the left, dyspnea on exertion, and mild blood-tinged sputum. He had no history of unusual bleeding, bruising, ear pain, sore throat, nasal congestion, nausea, vomiting, diarrhea, dysuria, numbness, tingling, focal deficits, rash, or recent vision problems. He had new-onset back pain over the last few months. He was a never-smoker, drank alcohol occasionally, and denied any substance abuse.

Computed tomography (CT) of the abdomen/pelvis with contrast revealed bilateral renal masses with retroperitoneal lymphadenopathy and bilateral pulmonary nodules. Fine-needle aspiration cytology of the left lung revealed malignant cells derived from mRCC. Urinalysis showed red blood cells, but cystoscopy was unremarkable. A complete blood count revealed anemia, consistent with the anemia of chronic disease observed with low iron but elevated ferritin. Chest X-ray revealed a resolved 4 mm pneumothorax in the left lower lobe, a postoperative complication from the biopsy.

He was diagnosed with a right renal mass with multiple pulmonary metastases, bilateral pulmonary embolism, anemia of chronic disease, GERD, dysphagia, gross hematuria, and non-sustained ventricular tachycardia. He was managed with heparin bridging for biopsy, omeprazole for GERD, ferrous sulfate for iron-deficiency anemia, metoprolol and simvastatin for coronary artery disease, and tamsulosin and finasteride for benign prostatic hyperplasia. Hematology-Oncology was consulted, and the patient was planned for outpatient follow-up for the management of renal cell carcinoma with pulmonary metastases. He was advised to follow up with his primary care provider for hemoptysis, cardiology for non-sustained ventricular tachycardia, and urology for gross hematuria. He was discharged in a stable condition.

After consultation and detailed discussion with Hematology-Oncology regarding the disease, prognosis, and treatment options, immunotherapy with ipilimumab and nivolumab was recommended. The patient consented to proceed with the therapy, and the plan was to administer nivolumab 3 mg/kg (240 mg dose, intravenous) and ipilimumab 1 mg/kg (76 mg dose, intravenous) on days one, 21, 43, and 64.

The patient remained under regular follow-ups with Hematology-Oncology. A CT scan on 8/20/2020 showed a very good partial response, almost near-complete response, to the combination therapy. The oncologist decided to continue with checkpoint inhibitor therapy with nivolumab 480 mg every four weeks while discontinuing ipilimumab due to the side effects experienced by the patient, including generalized pruritus and a mild rash on his back. Gabapentin was prescribed by the primary care physician for pruritus, suspected to be a medication side effect with a possible neuropathic component.

Despite no initial side effects, except for shingles, which were treated with Valtrex, the patient later developed generalized pruritus and pinprick sensations, not relieved by Benadryl or hot showers. The patient also reported long-standing throat irritation, unrelated to pruritus, which prompted an ear, nose, and throat evaluation. Meanwhile, the oncologist attributed the pruritus and hyperthyroidism to immunotherapy and decided to monitor the patient’s symptoms closely. However, as shown in the laboratory values in Table 1, the patient’s erythrocyte sedimentation rate increased to 120 mm/hour, CRP increased to 12.1 mg/dL, and he developed anemia with a hemoglobin level was 9.3 mg/dL. Transient vision loss was noted, raising suspicion for GCA. High-dose prednisone was initiated, and referrals to Rheumatology and Neurology were made for further evaluation. Despite a negative temporal artery biopsy, the patient’s symptoms and excessively high inflammatory markers led to a diagnosis of GCA by the rheumatologist. The patient was educated about GCA and advised to restart prednisone at a higher dose, tapering it down gradually.

**Table 1:** Inflammatory markers.

Investigation	Result	Normal range
Erythrocyte sedimentation rate – blood	120 mm/hour	0–13 mm/hour
Serum C-reactive protein	12.1 mg/dL	0–1.0 mg/dL
Hemoglobin	9.3 mg/dL	13.5–15.5 mg/dL

Due to the recent diagnosis of GCA, possibly associated with checkpoint inhibitor cancer therapy, the decision was made to hold off on nivolumab treatment and closely monitor the patient’s cancer status. The oncologist reviewed the latest CT scans, which showed stable disease from a cancer standpoint. The patient was advised to continue follow-ups with the rheumatologist and oncologist.

The case study presented here exemplifies the complex managerial choices encountered by patients afflicted with mRCC undergoing immunotherapy. Although nivolumab and ipilimumab initially elicited a positive response, the emergence of GCA, a severe irAE, necessitated the cessation of treatment. This case underscores the need for diligent monitoring of autoimmune ailments in individuals receiving checkpoint inhibitor therapy and stresses the requisite involvement of a multidisciplinary medical team in their care.

## Discussion

Extensive literature has established the remarkable efficacy of immunotherapy in managing malignant renal cell carcinoma. However, in this rare and unusual case, this form of treatment led to the manifestation of GCA, necessitating its cessation. This occurrence is an example of the potential side effects of immunotherapy for malignant renal cell carcinoma. Despite a positive patient response to nivolumab and ipilimumab therapy, the development of GCA was an unforeseen consequence that required immediate immunotherapy discontinuation.

Nonetheless, checkpoint inhibitor therapy modifies the regulatory pathways in T cells to enhance their ability to fight against tumors, resulting in significant progress in treating advanced cancers such as bladder tumors and renal cell carcinomas [7]. The initiation of therapy is based on crafting an optimal drug combination to optimize results, leading to a more substantial anti-cancer effect [8]. Although the efficacy of checkpoint inhibitor therapies in adults has been remarkable, a significant proportion of patients experience mild-to-moderate adverse reactions contingent upon the dosage, which is observed in over 70% of patients [9].

The importance of this particular case lies in its valuable contribution to our overall understanding of the potential adverse effects associated with checkpoint inhibitor therapy [10]. These inhibitors operate by modifying immune activation processes and may, on occasion, induce immune-mediated inflammation in different organs or tissues. This is a rare occurrence that is frequently difficult to diagnose, necessitating the utilization of high doses of immunosuppressive drugs and steroids [11]. In our case, the patient experienced an unfavorable inflammatory reaction resulting in GCA after receiving checkpoint inhibitor therapy. Consequently, it emphasizes the critical need for continued research and vigilance to enhance the safety profile of these treatments which show promise.

Neuro-ophthalmic complications have been reported in patients treated with checkpoint inhibitor therapies. In a systematic review, 0.46% of patients treated with checkpoint inhibitor therapy suffered from neuro-ophthalmic complications. Of these patients, 12.8% suffered from optic neuritis, 0.9% reported neuro-retinitis, while GCA was seen in 3.7% [12]. Patients who developed GCA secondary to therapy presented with identical idiopathic GCA symptoms, including visual abnormalities such as blurry vision and diplopia, scalp tenderness, and jaw claudication. One patient was also reported to have presented with a sudden onset of total vision loss, while another reported no visual abnormalities [13,14]. In the systematic review, three of the five cases who presented with GCA also developed polymyalgia rheumatica [15]. Most of these cases of GCA were treated with high-dose corticosteroids after complete cessation of checkpoint inhibitor therapy [13].

In our case, the initial side effects noted were pruritus and throat irritation, followed by hyperthyroidism. However, the first side effect that indicated the possibility of GCA was a transient loss of vision and a raised erythrocyte sedimentation rate. Kreuter et al. elaborated on a similar case of nivolumab-associated GCA. Similar to our case report, the patient presented with altered vision. However, contrary to our patient, the latter had significant scalp necrosis, tenderness, and a positive temporal artery biopsy [16]. A study by Xiao et al. concluded that though GCA secondary to checkpoint inhibitor cancer therapy is rare, GCA is generally manifested by headache, temporal artery tenderness, and diplopia, followed by a confirmatory biopsy [17].

As we explore the negative occurrences linked to cancer therapy utilizing checkpoint inhibitors, it becomes more apparent that this therapy’s efficacy in treating advanced-stage tumors has broadened its clinical scope. Nonetheless, an essential aspect of its usage depends on healthcare professionals’ comprehension of its possible unfavorable consequences for a patient’s well-being. Given the diverse range of side effects connected with these treatments, it is crucial to establish the ideal dose of anti-tumor therapy, which can achieve favorable outcomes and reduce detrimental impacts on the patient’s health.

Ensuring precise dosage is imperative for the timely detection of undesirable effects, a crucial aspect of enhancing patient outcomes. Timely recognition permits expedited implementation of immunosuppressive measures, which positively influence patient outcomes. The vigilant monitoring and management of adverse events can improve the safety and efficacy of checkpoint inhibitor therapy in treating advanced-stage tumors, thereby promoting favorable patient outcomes. Thus, healthcare providers must remain attentive to such events and promptly take appropriate action.

## Conclusions

When managing mRCC with checkpoint inhibitor therapy, clinicians must remain attentive to potential severe irAEs, including those as infrequent as GCA. This case highlights the delicate balance between treatment effectiveness and the unforeseen complications that these innovative therapies may bring. In such situations, an interdisciplinary approach that combines oncological, rheumatological, and primary care expertise becomes essential. Given the medical community’s continued exploration of the potential of immunotherapies, it is critical to record and sift through distinctive cases. Doing so will promote a more profound comprehension of these treatments while providing better patient counseling and comprehensive management strategies customized to individual patient needs in navigating modern cancer therapeutics’ complex realm.
